# Barriers and facilitators to the effective de-escalation of conflict behaviours in forensic high-secure settings: a qualitative study

**DOI:** 10.1186/s13033-020-00392-5

**Published:** 2020-08-02

**Authors:** Helena Goodman, Cat Papastavrou Brooks, Owen Price, Elizabeth Alexandra Barley

**Affiliations:** 1 Kate Granger Building, University of Surrey, Guildford, GU2 7YH UK; 2 Jean McFarlane Building, University of Manchester, Manchester, M13 9PY UK

**Keywords:** Aggression, De-escalation, High-secure hospital, Qualitative, Restrictive practices, Trauma, Violence, Behaviour change

## Abstract

**Background:**

Violent and aggressive incidents are common within mental health settings and are often managed using high-risk physical interventions such as restraint and seclusion. De-escalation is a first-line technique to manage conflict behaviours and prevent violence and aggression. There is limited research into the use of de-escalation in high-secure settings. This study investigated staff, patient and carer perspectives on the barriers and facilitators to using de-escalation for conflict behaviours.

**Methods:**

Semi-structured individual interviews (n = 12) and focus groups (n = 3) were conducted with eight patients, four carers and 25 staff members in a high-secure hospital in England. Interviews and focus groups were informed by the theoretical domains framework and were digitally recorded, transcribed verbatim and analysed using framework analysis and the COM-B behaviour change model.

**Results:**

Four themes and 15 sub-themes (barriers and facilitators) were identified. Themes related to capabilities (building relationships: knowing the patient and knowing yourself), opportunities (filling the void: challenges within the high-security environment; dynamic relationships) and motivation (keeping everyone safe). Strong staff–patient therapeutic relationships underpinned by trust, fairness, consistency and an awareness of the trauma-aggression link were considered key to successful de-escalation. Specific psychological and interpersonal skills including empathy, respect, reassurance, sincerity, genuine concern and validation of the patient perspective are needed to achieve this. Barriers related to the physical environment; organisational resources, practices and systems; staff traumatisation; hierarchical and punitive attitudes towards patient care, and an insufficient understanding of psychiatric diagnoses, especially personality disorder. It was apparent across themes that fear, which was experienced by both staff and patients, was a driver for many behaviours.

**Conclusions:**

This work has identified organizational and behaviour change targets for interventions seeking to reduce violence and restrictive practices through the use of de-escalation in high-secure hospitals. The potential for, and occurrence of, violence in such settings is high and leads to fear in patients and staff. The factors which promote fear in each group should be addressed in de-escalation training.

## Background

High-secure hospitals in the UK provide both custody and treatment for mentally disordered offenders considered to pose ‘a grave and immediate danger to the public’ [1]. All patients are detained involuntarily under the Mental Health Act (1983) and typically present with challenging behaviours, including violence and aggression [2, 3]. The ongoing demands, expectations and conflict inherent in inpatient treatment may provoke anger in forensic patients who may have limited skills to manage this [4], thereby increasing the readiness to react aggressively [5] The proportion of staff and patients who have been victims of, or directly involved in, violent or aggressive incidents in high-secure hospitals is considerable (36–82% of staff and 32–46% of patients) [6–8]. Furthermore, the prevention and management of conflict behaviours is costly [9]. Given the high proportion of violent and/or aggressive incidents in high-secure settings (0.89 incidents per patient per month) [6], the safe resolution of violent and aggressive behaviour is key.

A range of management strategies is available, including non-physical or interpersonal approaches (e.g. de-escalation) and restrictive interventions (e.g. tranquilising medication, seclusion and physical restraint) [10]. Due to their potential for physical and/or psychological harm to those involved in or witnessing their use, there is an international drive to minimise the use of restrictive interventions so that they are used only as a last resort [8, 11–16].

De-escalation aims to reduce violence and aggression, and hence avoid the use of restrictive interventions, by negotiating a mutually agreeable solution to the aggressor’s concerns [10, 17]. Non-provocative communication skills are used to gradually resolve potentially aggressive situations by redirecting the patient to a calmer personal space [18]. However, its use is complicated by the absence of a ‘best-practice’ method. A systematic review (*n* = 39 studies) reported significant heterogeneity in the content and duration of training courses incorporating de-escalation and a lack of clarity around the effectiveness of training [19]. There is also evidence of a lack of consensus and consistency in how the term ‘de-escalation’ is operationalised despite efforts to clarify this [20] and an insufficient understanding of de-escalation components [17].

Furthermore, evidence underpinning de-escalation practices and training is biased towards adult acute and psychiatric intensive care units. Equivalent data from forensic settings are lacking [21, 22] so there is limited understanding of the specific interpersonal and contextual factors that impact on effectiveness within these settings, and, in particular, within high-secure settings. For instance, a well conducted systematic review of violence prevention in inpatient settings [20] found only two studies in high-secure settings: one in the UK which was a questionnaire study [8] and a focus group study conducted in Australia [23]. Similarly, research has been predominantly conducted from a clinical perspective and patient and carer views have been neglected [17]. This is important considering contrasting perceptions between nurses and patients of violent incidents and responses to them [24, 25]. The present study, therefore, was conducted to explore the perceived barriers and facilitators to effective de-escalation from a high-secure patient, carer and staff viewpoint. The findings will contribute to developing an evidence based, theory-informed de-escalation training as part of a larger NIHR-funded project (HTA Project: 16/101/02).

## Methods

### Study design

A qualitative study using semi-structured interviews and focus groups was conducted. The COM-B (‘capability’, ‘opportunity’, ‘motivation’ and ‘behaviour’) behaviour change model [25] and the Theoretical Domains Framework (TDF) [26] informed both data collection and analysis.

### Setting

This study took place in a high-secure hospital in the UK. The hospital has 284 beds and provides inpatient care for adult males.

### Participants

#### Staff

Participants were recruited from three groups: frontline-clinical staff (nurses up to ward manager level and healthcare facilitators), the multi-disciplinary team (MDT: service managers, occupational therapists, psychologists, psychiatrists, social workers) and specialists in the prevention and management of violence and aggression (PMVA). A convenience sample (i.e. those present when the researcher was available) of sixteen frontline-clinical staff (nine healthcare facilitators and seven nurses) from each ward category (admission, high-dependency, assertive rehabilitation and intensive-care) were invited to participate in a de-escalation-themed focus group. Each staff member from the PMVA department (n = 9; this includes three who also work as part-time healthcare facilitators) was contacted via e-mail and invited by researcher (HG) to attend a de-escalation themed focus group. All members from the hospital’s MDT were invited to participate by the hospital’s Research Lead (a forensic psychiatrist) via email.

To ensure that participants were sufficiently experienced, only those who had worked in high-secure services for a minimum of 6 months were included.

#### Patients

Patients were recruited from one admission, one high-dependency and one assertive rehabilitation ward. The hospital has 15 wards, which are categorised into ‘admission’ (n = 3), ‘high-dependency’ (n = 4), assertive-rehabilitation (n = 7) or intensive-care (n = 1). To ensure maximum variation in patient experience of de-escalation, the hospital’s Research Lead recommended that researchers recruit patients from one of each ward category except for intensive-care. This was based on the severity of illness symptomatology, likelihood for aggression and lack of capacity amongst this patient population. Prior to study engagement, the ward’s Responsible Clinician (RC) was contacted to establish each patient’s capacity to give informed consent and to request permission for them to be approached. Only patients deemed by the RC to have capacity to understand the study’s aims and provide informed consent were approached. Twenty-eight prospective participants (16 from assertive rehabilitation; six from high-dependency and six from admission wards) were then approached and informed by the researcher about the purpose of the research, which was provided in a participant information pack. Of these, eight agreed to participate.

#### Carers

Carers were recruited through convenience sampling. The study was presented at an on-site carer’s forum, which approximately 15 carers attended. Carers who expressed an interest in participation were contacted via telephone or e-mail by the researcher (HG). Anyone describing themselves as a carer was eligible to participate.

### Procedure

Prospective participants were provided with a participant information pack which explained the purpose of the study, gave information about the research team and detailed the potential risks and benefits of taking part. Opportunity was provided to ask questions. For those agreeing to participate, a meeting was arranged to obtain consent and to conduct the interview (patients and carers) or focus group (staff). Interviews were face to face for patients and some carers, other carers opted to be interviewed by telephone. Separate focus groups were conducted for the three professional groups (i.e. one focus group for MDT; one for PMVA; one for frontline ward staff). Recruitment processes are detailed below and sociodemographic characteristics of frontline clinical, MDT and PMVA staff are detailed in Tables 1, 2 and 3, respectively. All participants were made aware that the interviewer (HG) also works as a bank healthcare facilitator in the same hospital. A favourable opinion for the study was provided by the South Yorkshire research ethics committee on 03/2018 (REC number: 18/YH/0035) as one component of a National Institute of Health Research-funded study to develop de-escalation training: the Enhancing de-escalation techniques in adult acute and forensic units: development and evaluation of an evidence-based training intervention (EDITION) study (HTA Project: 16/101/02).

**Table 1:** Sociodemographic characteristics of frontline clinical staff

	*n*
Gender	
Male	3
Female	2
Age (years)^a^	
18–29	3
30–39	–
40–49	–
50+	2
Occupation	
Nurse	2
Healthcare assistant	3
Clinical experience (years)^b^	
< 12 months	–
1–5 years	3
6–10 years	–
11–20 years	–
21+ years	2

Age (years)^a^: M = 38.8; SD = 16.90

Clinical experience (years)^b^: *M *= 11.0; SD = 11.5

**Table 2:** Sociodemographic characteristics of MDT

	*n*
Gender	
Male	1
Female	10
Age (years)^a^	
18–29	3
30–39	1
40–49	2
50+	5
Occupation	
Psychiatrist	4
Psychologist	4
Occupational therapist	2
Psychology student	1
Clinical experience (years)^b^	
< 12 months	1
1–5 years	3
6–10 years	2
11–20 years	2
21+ years	3

Age (years)^a^: *M *= 41.5; SD = 14.52

Clinical experience (years)^b^: M = 14.4; SD = 12.28

**Table 3:** Sociodemographic characters of PMVA specialists

	*n*
Gender	
Male	7
Female	2
Age (years)^a^	
18–29	–
30–39	1
40–49	5
50+	3
Clinical experience (years)^b^	
< 12 months	–
1–5 years	–
6–10 years	1
11–20 years	3
21+ years	5

Age (years)^a^: *M *= 46.0; SD = 6.32

Clinical experience (years)^b^: *M *= 20.9; SD = 6.52

### Data collection

All participants provided information on their age, gender and ethnicity. Clinicians were also asked about their role and experience. Patients were questioned regarding their primary diagnosis’ current length of stay in hospital and interventions they had received. Carers were asked to provide this information regarding the person they cared for.

Staff focus groups and carer and patient interviews were guided by a topic guide (see Additional files 1, 2 and 3, respectively) informed by the TDF [27]. The TDF covers a set of domains comprising evidence-based factors influencing behaviour change, such as knowledge, beliefs about the consequences of the target behaviour, social influences such as the attitudes of close others, and the environmental context. The framework allows researchers to explore the content of each domain with respect to the behaviour of interest, in this case the management of potential incidences of violence and aggression.

Focus groups were facilitated by EB with support from HG; interviews were conducted by HG. Discussion was structured around participants’ expectations of and experiences and perspectives on the use and effectiveness of de-escalation techniques. The EDITION study definition for de-escalation techniques was read out verbatim prior to the commencement of each interview and focus group: “verbal and non-verbal skills and strategies to reduce aggression without methods including physical restraint, medication or seclusion”. Six conflict behaviours were specified: aggression, self-harm, medication refusal, rule-breaking, absconding and suspected or proven alcohol/drug use. Additional topics were pursued when raised. All discussions were recorded using a security authorized digital recorder and transcribed verbatim.

### Data analysis

Analysis was informed by the COM-B model of behaviour change [26]. Framework Analysis comprising data familiarisation, indexing, charting and mapping and interpretation [28] was adopted and facilitated using NVivo 11 software [29]. As Framework Analysis permits the use of both inductive and deductive coding, researchers were able to include both a priori (e.g. the TDF determinants) and emergent codes. This method ensured that important themes were not lost through deductive data analysis.

All authors read and familiarised themselves with the transcripts. The authors represent a wide range of disciplines and include those (OP, HG) with long term experience of working clinically in high-secure settings, furthermore, the research was conducted in collaboration with the wider EDITION project MDT. Each transcript was subject to line-by-line analysis, in which verbatim data were coded in the relevant TDF domain. To ensure coding reliability, transcripts were analysed by one researcher (HG) and 20% independently coded and reviewed by another (EB). Discrepancies were resolved through discussion. Additional codes and categories identified in the familiarisation and indexing stages were added to the framework. The charting and mapping stages involved generating a framework from the codes using the Framework function of NVivo11 [29] with columns representing the TDF domains and codes and rows representing cases.

Each transcript was subject to further interrogation, in which verbatim data were summarised and relevant cells populated with summarised data, including key quotations. The ‘create summary link’ function was used to link data summaries with verbatim data enabling recall at later stages of analysis. This process involved data immersion and therefore allowed for greater refinement or modification of the generated themes and sub-themes. A detailed matrix of data, which was discussed and agreed within the entire team, was then produced to highlight key themes, alongside de-escalation barriers and facilitators within each TDF domain and mapped to the COM-B model [26].

## Results

There were 37 participants (12 interviews; three focus groups). Twenty-five staff participated in three focus groups (duration range 56–186 min). All 16 frontline-clinical staff invited to attend their respective focus group agreed to participate, however only 5 could attend due to insufficient ward staffing on the day. The frontline-clinical staff focus group lasted 1 h and 28 min.

All members of the MDT attended their respective focus group, which lasted 58 min.

All PMVA specialists (n = 9) within the department attended their respective focus group, which lasted 3 h and 07 min.

Eight patients participated in interviews, which lasted between 24 and 58 min (mean = 36 min) (Table 4).

**Table 4:** Socio-demographic characteristics of patients

	*n*	%
Age (years)^a^		
18–29	2	25
30–39	4	50
40–50	2	25
Ethnicity		
Black or British—Caribbean	1	12.5
Black or British—African	1	12.5
White-British	6	75
Received interventions		
Physical restraint	7	87.5
Compulsory medication given by injection	4	50
Seclusion	8	100
PRN medication	6	75
Increased observation	7	87.5
Self-reported diagnosis		
Psychotic disorders	2	25
Dual diagnosis (psychotic and personality disorder)	2	25
Dual diagnosis (Personality and mood disorder)	1	12.5
Multiple diagnoses (personality, mood and anxiety disorder)	2	25
Multiple diagnoses (personality, mood, psychotic and anxiety disorder)	1	12.5
Length of stay in hospital		
< 12 months	1	12.5
1–5 years	3	37.5
6–10 years	1	12.5
10+ years	3	37.5

Age (years)^a^: *M *= 35.8; SD = 7.14

Two carers participated in face-to-face interviews and two in telephone interviews (Table 5), which lasted between 31 and 42 min (mean = 38 min).

**Table 5:** Socio-demographic characteristics of carers

	*n*	%
Gender		
Female	4	100
Age (years)^a^		
40–49	2	50
50+	2	50
Ethnicity		
Black or Black British—Caribbean	1	25
White-British	2	50
Mixed—White and Black Caribbean	1	25
Details for person cared for		
Age (years)		
18–29	1	25
30–39	1	25
40+	2	50
Ethnicity		
Black or British Caribbean	1	25
Mixed—White and Black Caribbean	1	25
White-British	2	50
Diagnosis category		
Psychotic disorders	2	50
Dual-diagnosis (psychotic and personality disorder)	2	50
Received interventions		
Physical restraint	3	75
Compulsory medication given by injection	2	50
Seclusion	3	75
PRN medication	1	25
None	0	0

Age (years)^a^: M = 58; SD = 5.47

Four themes and 15 subthemes (barriers and facilitators) across eight domains of the TDF [27] and spanning all COM-B areas [26] were identified. These are summarised in Table 6 and presented below with supporting quotes identified by participant number (e.g. P1), gender and participant group/role. The ‘memory, attention and decision-processes’; ‘physical skills’; ‘goals’; ‘beliefs about consequences’; ‘beliefs about capabilities’ and ‘optimism’ and ‘reinforcement’ domains of the TDF did not emerge from the data as particular targets for behaviour change in this setting. Participants overwhelmingly discussed de-escalation in the context of managing violence and aggression, rather than for self-harm, absconding, rule-breaking, medication-refusal, suspected or proven alcohol/substance misuse.

**Table 6:** Barriers and facilitators to effective de-escalation using the TDF [27]

COM-B	Theme	Sub-theme (barriers and facilitators)	TDF
Capability	Building relationships: knowing the patient and knowing yourself	Creating an authentic relationship across social distance: rapport versus compassionate engagement	Psychological skills
		An individualised de-escalation approach	Psychological skills
		Knowing about the patient: stigmatising attitudes	Knowledge
		Patient trauma	Knowledge
		Managing emotions	Knowledge
		An ethos of positive risk-taking and least restrictive practice	Behavioural regulation
Opportunity	Filling the void: challenges within the high-secure environment	Organisational resources	Environmental context and resources
		The ward environment	Environmental context and resources
	Dynamic relationships	Power and control over patients	Social influences
		A supportive and collaborative workforce	Social influences
		Gender and de-escalation	Social influences
Motivation	Keeping everyone safe	Early intervention: recognising warning signs	Social/professional role
		De-escalation: an inbuilt and ongoing process	Intentions
		Staff traumatisation	Emotion
		Boundaries: the function of ‘consistency’	Social/professional role

### Capability

#### Theme: Building relationships: knowing the patient and knowing yourself

i Creating an authentic relationship across social distance: rapport versus compassionate engagement

The dominant view among all participants was that a strong therapeutic relationship is most important, with successful de-escalators being perceived as sincere and credible in how they relate to patients:

*“I think what’s helpful is [staff] being compassionate, like understanding… understanding why you do what you do” (P33, patient, high*-*dependency ward)*

*“It is more just developing a relationship with the patient… there were two members of staff when I was having a visit on the ward and I could not believe how one of them was the ward manager. I couldn’t believe how empathic he was to my brother when he was talking to him. He was reassuring my brother that he understands his perspective. The words he was using and the rapport… you could see there was some kind of mutual respect between the two of them” (P35, carer)*

*“if you are switching something on and it’s not something that you believe in yourself people pick up on that… it is a more long*-*term thing than just an acute attribute you switch on and off because someone has kicked off” (P25, female, psychologist)*.

This acknowedgement of authentic staff–patient relationships as foundational to de-escalation is complicated by fears, especially amongst ward-based staff, about becoming close to patients in a way that could create co-dependency and become “burdensome” for them (P14, male, nurse). Whilst staff members across groups felt that it was important to maintain social and emotional distance from patients; there was recognition that a detached or cognitive form of empathy was necessary for understanding patients, however affective empathy (or sympathy) which necessarily involves emotional engagement with patients was seen by ward staff as a risk factor for boundary violations:

*“You can learn empathy as long as it does not show as sympathy […] because if you have walked a mile in their shoes you are going to start to feel sympathy, if you have shared the experiences and that’s when boundaries are crossed and so on and so on […] so if you start showing sympathy then it can be, that gets over a long period of time it gets picked at and picked at until you have shared information that you shouldn’t have shared (P11, male, healthcare*-*assistant)*

In the context of this perceived conflict between staff’s need to maintain distance from patients and to build a credible relationship necessary for de-escalation, frontline staff focused on the importance of having ‘rapport’ with patients; a less emotionally engaged style of interaction characterised by staff as a combination of ‘banter’, humour and doing ‘day-to-day tasks’ together.

Although the concept of ‘rapport’ as key for de-escalation was consistently emphasized across the different staff groups, patients reported that ward staff attempts to create this superficial form of familiarity were a frequent cause of staff–patient conflict, and the resultant use of restrictive practices. The disjunct between the power-imbalance inherent in staff–patient relationships, and the more informal style of interaction staff saw as constituting rapport-building was often so jarring to patients that they interpreted it as a deliberate provocation:

*“Sometimes it can feel like they are rubbing things in. They will come in telling you stuff about their lives outside and, you know, trying to crack jokes with you which are kind of inappropriate” (P30, patient, assertive rehabilitation ward)*

*“Especially when staff will go ‘how’s your mum DELETED NAME, you know I don’t see my mum, why do you ask you know what I mean or when are you next seeing your mum, they know I don’t see her so them sort of things are kind of insensitive and it’s not like they are doing it accidentally they know.” (P28, patient, assertive rehabilitation)*

In contrast to staff anxieties about over-identification, patients emphasised the need for staff to be both emotionally present (e.g. expressing genuine concern) and actively engaged (e.g. frequent one-to-one conversations) in order to promote trust and respect. Psychology staff were often named by patients as most likely to elicit trust, as they frequently engaged emotionally with patients through the use of questions which communicated sincere interest and compassion:

*“Talking about my thoughts when I’m low, thoughts when I’m high. Which leads on to other things, other conversations, they’re the right conversations.” (P29, patient, assertive rehabilitation ward)*

ii. An individualised de-escalation approach

The value of a patient-centered approach to de-escalation was highlighted across all groups on the basis that an approach successful for one patient (e.g. to be given time and space) may not be for another. Staff and carers suggested that this is achieved by ensuring that each shift includes staff with different interpersonal styles and skills.

*“if you are doing your rota make sure you’ve got a combination of staff that can work on different things that are able to handle different situations and stuff” (P34, carer)*

Frontline clinical and PMVA staff valued advance directives, which involve the patient in collaborative de-escalation planning, to identify (i) potential triggers and early warning signs of aggression and (ii) preferences for de-escalation/management.

*“Advance directives prevent it [aggression] going to that stage [violence] and setting good de*-*escalation strategies in place prior to it to prevent it happening [violence]” (P5, female, PMVA instructor)*

iii. Knowing about the patient: stigmatising attitudes

Staff reflected on how labels such as “mad, bad or sad” (P1, male, PMVA instructor) were often used to relate to people with psychotic, personality and depressive disorders respectively, resulting in dichotomous attitudes towards patients seen to have “illness-related” or “non-illness-related” aggression. Frontline and PMVA staff discussed how aggression in the context of psychosis was typically attributed to acute mental distress and elicited more sympathetic and less punitive responses compared to patients with a diagnosis of personality disorder:

*“Someone who is suffering from psychosis… staff tend to sympathise…. so if they (patients) say something in the heat of the moment and its quite personal… staff understand they are unwell. But someone who might say the same but who staff perceive as just ‘bad’, staff take it quite personally… it becomes an emotive situation” (P1, male, PMVA instructor)*

The underlying assumption that the behaviour of patients with a diagnosis of personality disorder was attributable to deliberate and malicious intent, not distress, meant that such behaviour was seen as “manipulative” and “demanding” by these staff, leading to unsympathetic responses. The combination of a false attribution of control and a negative moral judgment about patients is exemplified in the concept of ‘team-splitting’, where differences in particular staff–patient relationships are interpreted as a deliberate attempt to undermine the unity of the ward staff team:

*“With my ward being personality disorder, one of the biggest challenges that we have is team splitting and having the patients pay a lot of attention to some staff members in a positive way and then in a negative way to others, deliberately putting the team at loggerheads” (P12, female, healthcare facilitator)*

Whilst no such distinction was made by the MDT between “illness-related” and “non-illness-related” aggression amongst patients with psychotic and personality-disordered patients, there was a belief that de-escalation approaches ought to be tailored according to patient diagnosis:

*“It depends on the context. If it [a violent/aggressive incident] concerns someone with a personality disorder or someone who is depressed or anxious, you tailor it to the patient’s needs. With personality disorder you want to put the responsibility back with the patient” (P15, female, psychologist)*

Participants from all groups felt that in order to improve de-escalation practices, interventions to enhance these skills would first need to modify staff knowledge and understanding of personality disorders and change attitudes.

vi. Patient trauma

All participants discussed the need for de-escalation approaches to be underpinned by an acknowledgement that most patients in high-secure settings will have experienced significant lifetime trauma and adverse childhood events (ACEs). Staff and carers spoke about the value of staff reframing aggressive behaviours in terms of a survival function in response to situational and/or relational triggers, especially among individuals with a personality disorder. Patients felt that heavy-handed approaches to escalating aggression and the over-use of alarm bells may cause fear and retraumatise victims, thereby increasing aggressive behaviours:

*“Being surrounded by like 5 members of staff when you are feeling agitated. I don’t think that helps …… it puts peoples’ back up and makes people go into defence mode. A lot of patients have had traumatic lives, had things done to them” (P28, patient, assertive rehabilitation ward)*

v. Managing emotions

The ability of staff to regulate their emotions whilst engaging in de-escalation was seen by all groups as essential. Specific communication skills were noted as important, in particular speaking in a calm and controlled manner; giving simple and direct instructions; maintaining a composed exterior with open and non-threatening body-language. Overt displays of anxiety, fear and frustration in staff were widely perceived to escalate aggression. One patient described how visible anxiety in a staff member triggered intrusive thoughts and some distressing ambivalence about his own capacity for self-control, e.g.:

*“The fact that he was clearly letting me know that he feared I was going to do something made me question whether I was gonna do something.” (P28, patient, assertive rehabilitation ward)*

However, staff reflected on the difficulties of remaining emotionally neutral in response to personally-directed and sustained verbal abuse. Knowing when to ‘step back’ *(P23, female, Psychiatrist)* was important in this situation.

*“(It) is about how personal it feels…depending on the nature of what people say, what is personally tolerable and not tolerable to you… that’s a big issue. Its particularly difficult for people who are consistently on the receiving end of really personal aggression” (P23, female, Psychiatrist)*

The negative impact on staff anxieties of emotive language in nursing notes, handovers and debriefs, and resulting impact on capacity for self-regulation during de-escalation, was also noted:

*“The doctor said ‘I just want to point out that this individual likes to fight and he’s very capable’. I sat there and I thought ‘wow, the anxieties that you’ve just released right at the end of the debrief’…It was really frustrating” (P5, female, PMVA instructor)*

Staff agreed that ‘debriefs’ and ‘safety huddles’ are important safe areas in which they can express emotions and seek support from peers to regulate their emotions and thereby improve de-escalation practice:

*“It is so important to have that debrief and ask how everybody feels about it (incident) because if you go home without talking about it, it festers overnight” (P12, female, healthcare*-*facilitator)*

iv. An ethos of positive risk-taking and least restrictive practice

There was a clear emphasis on positive risk taking and least restrictive practices such as de-escalation at all levels of the organisation. However, frontline staff described a lack of clarity surrounding the principles of least restrictive practice and positive risk taking and a top-down organisational blame culture that created fear, confusion and unsafe conditions for effective de-escalation; a matter that was not raised by MDT or PMVA professionals:

*“If someone had an incident and was secluded then … we would maybe stop them from going off ward the day after … because ‘you did something wrong, therefore you shouldn’t get the nice thing you wanted to do’, but then that seems being very punitive so then ok so you were secluded yesterday but you’re ok today we’ll send you off to do something. And then something happens in that area…. then ‘why did you let them go, they were secluded yesterday?’” (P13, female, healthcare*-*facilitator)*

### Opportunity

#### Theme: Filling the void: challenges within the high-secure environment

Participants from all groups perceived the confinement and rules which are considered essential for risk management within high-secure settings to be barriers to de-escalation. Staff members often referred to the highly regimented nature of high-secure settings, where even the most basic items such as toothbrushes are monitored, which seemed to create some pessimism among staff about the opportunities for enhanced de-escalation, a pessimism that importantly wasn’t raised by patients.

*“if we wrote our wish list, what we would describe is a low*-*secure hospital, but we are not…. we take lot away from patients. They live with very little, almost no, privacy and no access to some of the most basic things that we take for granted” (P21, female, Psychologist)*

i. Organisational resources

A commonly identified barrier to effective de-escalation was insufficient staff time and a lack of meaningful activity for patients. A shortage of ward staff alongside perceived excess bureaucracy was considered to cause an over-reliance on restrictive interventions. Participants reported the frequent cancellation of off-ward activities caused by lack of staff. Staff identified enforced idleness as a major cause of avoidable conflict and that it limited opportunities to redirect patients to activities for the purposes of de-escalation. There were concerns raised over the quality as well as the extent of the staffing. Owing to problems with recruitment, staff with little or no mental health knowledge or experience were employed, and participants identified this as a key barrier to de-escalation:

*“They have no experience at all of working in a psychiatric hospital, no training and don’t know about mental illness or personality disorders… then we’re expecting them to go on to wards and be able to de*-*escalate.” (P21, female, Psychologist)*

ii. The ward environment

Patient views of the ward environment varied depending on where they were located. Those on assertive rehabilitation tended to have favorable impressions, highlighting the quiet, low-stimulus environment as conducive to de-escalation. Physical environments that had a range of accessible areas and activities (e.g. cooking) to use during times of distress were highly valued. Patients valued the relative freedom on these wards:

*“I think it’s better because you can do more stuff. You can cook, you can make hot drinks whenever you like, stay up later. It makes you feel a bit more free” (P28, patient, assertive rehabilitation ward)*

In contrast, open-ward layouts were often likened to “fishbowls” with nowhere to “escape”. Patients felt these environments reduced their dignity. Specifically, when incidents were witnessed by their peers, shame was experienced. Moreover, the close proximity of bedrooms can mean that de-escalation interactions aren’t confidential. The restrictions of living in a confined space for a long period of time with other volatile individuals left patients feeling that aggressive behaviour was sometimes inevitable and that this hampered staff de-escalation capability substantially:

*“In (open*-*wards)… It’s all like everyone is in everyone’s business and personal space all the time. Here (assertive*-*rehabilitation) there are actually places you can go to get away and go to the quiet room and you can have time out and stuff like that” (P28, patient, rehabilitation ward)*

#### Theme: Dynamic relationships

i. Power and control over patients

Carers and patients felt that some staff were preoccupied with maintaining custody, control and setting unnecessary limits on patient behaviour rather than on providing the therapeutic, psychosocial treatment of which de-escalation is a mainstay.

Some patients felt that de-escalation techniques are not used because certain staff rely on restrictive practices to assert their dominance, citing the use of coercive staff behaviours in the context of punishment and refusal to comply. On the contrary, some patients and carers felt that there were staff who deliberately provoked patients in order to elicit a reaction which would justify the use of restrictive interventions.

*“There are some who enjoy it. You know, you get the odd ‘policeman’ who enjoys the ego, who is corrupt…… It’s power* - *there are certain people who are not good when they are given power” (P36, Carer)*

Some staff felt that patients could develop an impression of individual staff members as especially coercive because those individuals, almost exclusively males, were over-relied upon for involvement in aggression management interventions. This could render patients less likely to respond positively to these staff members during escalations:

*It’s generally the same people who get picked all the time, they are always one step behind in building that relationship with that individual (patient)…they associate that staff member as being the one that will restrain you” (P14, male, nurse)*

ii. A supportive and collaborative workforce

Participants across staff groups perceived that collaboration across the MDT was an essential facilitator of de-escalation. This included formalised opportunities (e.g. Care Programme Approach meetings) to promote a shared understanding of the patient’s diagnosis, triggers and personal difficulties. A supportive, transparent and proactive approach to care planning was particularly valued in terms of increasing insight into difficulties and how to support change, fostering motivation and decreasing the likelihood of inconsistency in de-escalation approaches.

*“Psychology has moved to a much more active role within clinical teams. We have more opportunities to interface with the managers and the team leaders through team meetings and CPAs…we share ideas and work with these ideas and come to suggestions that might be helpful and tolerable.” (P15, female, Psychologist)*

Staff described the importance of feeling supported by management structures to promote an ethos of motivation, confidence and resilience within ward teams which they felt supported de-escalation. A useful tool in this respect was having frequent supervision that promoted openness, validated emotional distress and avoided penalising staff for taking sick leave. However, this was not always felt to be the case; the use of the ‘Bradford’ sickness-monitoring system, in particular, was considered by frontline staff to be punitive and to result in anxiety and stress that was not regarded as helpful psychological preparation for de-escalation:

*“When you go sick you lose your enhancements, you end up getting paid less, your Bradford Score goes up then you get pulled into meetings…you then get worried and you start working and the next thing you know you have a cold and you drain yourself even further. It’s a massive snowball effect” (P14, male, nurse)*

iii. Gender and de-escalation

The impact of staff gender on de-escalation was discussed by staff and patient participants, both in terms of the importance of a balanced gender mix, and the relationship between gender and perceived threat. Patients reported feeling powerless and scared when their behaviour was escalating. Feelings of safety were enhanced by the presence of staff they could trust; patients reported that such persons were often, though not always, female. This was partially attributed to women’s perceived enhanced de-escalation skills, and that women were also seen as less dominant or threatening, reducing patient’s fear and sense of powerlessness:

*“This comes back to again when they treat males and females differently they don’t expect a threat to come from a female they expect it from the males, so they are always on guard for most males.” (P13, female, healthcare assistant)*

Despite this, male staff members continue to be relied on by colleagues for their involvement in managing aggressive and violent incidents, even in instances where staff know that patient is likely to respond poorly to de-escalation attempts by male staff members:

*“I knew from previous experience that this individual doesn’t work very well with males. I brought it up and a [male] colleague disagreed with me, so I said “do you have a good rapport with this patient?”. He said, “no he has threatened to kill me”. I said “right so we won’t be using you then!”. I said “what about that chap over there?” and he said “oh no he’s been threatening to hurt him”. Then I said “What about that gentleman?”, and he said “no he’s made threats to him”. I said “right so we will be using the females”. They [females] managed to de*-*escalate the patient and he [patient] put all the items he damaged to the back of his room.” (P1, male, PMVA instructor).*

However, the perception of female staff members as more vulnerable often elicited feelings of over-protection amongst both patients and male colleagues, which could escalate situations of potential conflict:

*“A lot of the patients would say something like ‘if that patient gave you trouble I would sort them out for you’. You have to say, ‘no don’t do that I’m fine don’t worry about me’. They can be quite protective particularly of female staff because they see us as being more vulnerable” (P13, female, healthcare facilitator)*

### Motivation

#### Theme: Keeping everyone safe

i. Early intervention: recognising warning signs

Most participants believed that the best way of avoiding a cycle of escalation, aggression and containment was to prevent it through risk-assessment. Behaviours such as clenched fists, gritted teeth and pacing were highlighted as useful indicators for staff assessing the urgency of early intervention.

Patients and carers commonly reported that staff are too reactive to escalating situations, suggesting that there is more scope for the use of de-escalation techniques. There was agreement that the first step should be for staff to ask the reason for escalating behaviour, through adopting a gently enquiring style whilst avoiding preconceptions about the causes. Participants across all groups recommended greater time and space be offered and a greater tolerance of escalated behaviour, including voicing frustration through shouting or exercise.

*“When you see a patient who looks distressed, you should try and calm them down before it gets worse….. even if it feels hard to communicate with them at the time because they are speaking in a certain way, just approach them slowly, don’t rush, don’t be too strong, be soft and settled and try and understand how he is feeling.” (P26, patient, admission ward)*

ii. De-escalation: an inbuilt and ongoing process

A dominant view across all participant groups was that de-escalation is a daily, ongoing process and “way of being” rather than a simple skill. It was felt that de-escalation techniques should not be considered in isolation from other aspects of staff–patient relationships and processes that help maintain a safe and therapeutic environment. These include ensuring a thoughtful, open and consistent therapeutic milieu; a safe environment and a constant vigilance to the patient’s triggers and vulnerabilities:

*“We see de*-*escalation as diffusing an actual incident whereas we try to say to staff ‘you work a whole shift, think of all the moments when you’ve being able to avoid, distract, diffuse’. You know, they’re de*-*escalating all the time…” (P2, male, PMVA instructor)*

iii. Staff traumatisation

Staff across groups identified how the resultant trauma from being a victim of, or witnessing, an assault was a key barrier to effective de-escalation:

*“I think that a real challenge for an organisation where staff are battered verbally, emotionally and sometime literally physically on a day*-*to*-*day basis. To keep coming back and keep trying to take the heat out of a situation again and again, when you have been at genuine risk yourself is an enormous ask of people” (P23, female, Psychiatrist)*

The consequent emotional detachment and numbing as a result of trauma was perceived as being to some extent adaptive in enabling staff to continue to work in environments which were traumatising:

*“I think the trouble is if you have been seriously injured you shut down your feeling abilities, it’s the only way you can come back to work” (P23, female, Psychiatrist)*

However, emotional reactions to trauma, specifically fear, were felt, across all participant groups, to affect staff perception of patients, making them less optimistic about engaging in de-escalation and potentially resulting in the pre-emptive use of restrictive measures:

*“If staff are burnt out then it’s going to affect the way they perceive that individual” (P15, female, Psychologist)*

*“Whether the staff is aware of what is actually going on inside of them or whether their whole thing is ‘well I’m feeling something I’ve got to put it on the patient, I know how to deal with them’ […] rather than consider; ‘am I kind of putting too much on the patient?’ […] the issue is not the patient it’s actually me!” (P34, female, carer)*

iv Boundaries: the function of ‘consistency’

Discussion of staff consistency in boundary and behavioural limit-setting featured prominently across all participant groups as an important aspect of de-escalation. There were some staff who erroneously viewed boundary-setting as a de-escalation component and other (generally more experienced staff) who identified boundary-setting as a key barrier to de-escalation. Broadly, staff expressed the view that inconsistencies in rule and policy application and differences in conflict resolution styles could be barriers to de-escalation. However the value of consistency was predominantly explained by staff in terms of preventing patients from ‘pushing boundaries’ in order to deliberately undermine the staff team. Staff described consistent boundary maintenance as a tool for maintaining ingroup cohesion in the face of a patient group who were assumed to be hostile, rather than the therapeutic, emotional or even safety function this practice addressed:

*“Some will push boundaries with one staff member over another… so you will find that they play staff off against each other. If one member of staff says ‘no’ they will try a different member of staff that might say ‘yes’. It’s making sure that everyone is on the same page really” (P12, female, healthcare facilitator)*

*“Some (patients) will push boundaries with one staff member over another” (P14, male, nurse)*

Patients described the importance of staff consistency in terms of not wanting themselves to be accused by staff of “pushing boundaries”, particularly in cases where rules for staff and patients were markedly different:

*“If staff come in and you give them a compliment sometimes they can take that the wrong way…someone (patient) can come out of their room and staff will say ‘Oh that’s a nice shirt you have on today’ and that’s fine but then you can have a member of staff come in and you can say ‘Oh you’re looking nice today’ and they say ‘You can’t say that’. Why not? It’s just being normal, you know what I mean? Just giving a compliment, they say that’s pushing boundaries. …that can happen quite often” (P30, patient, assertive rehabilitation ward).*

However in situations of conflict, PMVA instructors acknowledged the importance of being flexible in rule and limit-setting with patients:

*“So not to be punitive in approach* - *‘you will do it and you’re going to do it this way’* – *so, if I thought I could resolve the situation by getting a patient a cake or something that I thought would give me a favourable outcome then within the realms of what you can do; then I would do that” (P3, male, part*-*time PMVA Instructor)*

## Discussion

This is the first qualitative study to address staff, patient and carer perspectives on capabilities, opportunities and motivation required to employ de-escalation in a UK high-secure forensic setting. TDF [27] domains that appear to be key to the use of de-escalation behaviours in this setting are: psychological skills, knowledge, behavioural regulation, emotion, environment, social influences, social and professional role and intentions (see Table 6). Although there was consistency across all participant groups on several key barriers and facilitators to de-escalation (such as the importance of staff–patient relationships) important differences were also present (for example in the types of communication styles perceived to be appropriate). Similarities with findings from studies in other mental health settings were also found, but issues perceived as unique to the high-secure setting were raised.

A dominant theme across accounts was the importance of the therapeutic relationship in the effective management of violence and aggression. Secure attachments are often absent in the lives of forensic patients who commonly have a history of ACEs marked by separation, loss, neglect, emotional and/or physical abuse [30–32]. However, the healing potential of relationships is well-established [33]; the extended length of stay in high-secure settings of patients compared with in other settings [34] may provide greater opportunity for the building of strong relationships. The value of therapeutic relationships was recognised by all participants but there were key differences in staff and patient views of the important components of these relationships. Consistent with previous literature [35], nursing staff described relying on more superficial forms of interaction such as joking or ‘banter’ to a greater extent than other professionals in building relationships. They also emphasised the importance of maintaining social distance from patients, which was felt necessary for emotional self-protection and owing to a perceived relationship between emotional closeness, boundary violations and resulting security breaches.

Patients, however, consistently reported a preference for relationships where staff were emotionally available and engaged them in a deeper and more authentic form of interaction. These findings echo those reported elsewhere in the literature [36]. Patient discomfort with nursing staff ‘banter’ was identified decades ago in the service user literature, with patients experiencing social pressure to reciprocate this form of humour, even in situations where they may not feel comfortable doing so [37]. In the same way, our findings indicate that high-secure patients view this humour as unacceptable, experiencing this form of interaction as antagonistic and humiliating. Nursing staff should be mindful of the power dynamics in their relationships with the patients, and the possibility that a patient’s apparent acceptance of this interactional styles may not reflect their actual feelings and experiences of it.

This study, as in others worldwide [38–40], noted the role of historical trauma and ACEs in the occurrence of violence and aggression and the re-traumatising impact of some conflict containment interventions such as seclusion and restraint. It is widely acknowledged that forensic patients have experienced exceptionally high levels of trauma. Reports suggest that between 95 and 100% of high-secure patients have experienced significant lifetime-trauma, often with multiple types of abuse [41, 42].

Trauma-informed approaches (TIAs) to care address the impact that trauma has on individuals’ worldview and interrelationships [43] and are based on the understanding that behaviours related to trauma are coping strategies developed to buffer against past traumatic experiences [44]. Through enhancing trauma awareness, a TIA aims to avoid staff and patient re-traumatisation by empowering individuals in decision making, safety, trustworthiness, choice and collaboration whilst building on personal strengths and skills [44]. In the context of high-secure hospitals, a TIA may serve to enhance de-escalation effectiveness by reframing conflict behaviours in terms of their survival function; encouraging care providers to address the underlying and unique needs of forensic patients; and countering the perception that patients, especially those with personality disorders, are manipulative, attention-seeking or destructive and decrease the use of restrictive practices. An intervention study would be needed to test this. Encouragingly, a narrative review [43] of trauma-informed mental healthcare interventions (*n* = 8 studies) in a range of mental health settings across the USA identified a reduction in seclusion, post-traumatic stress symptoms and general mental health symptoms, increased coping skills, improved physical health and shorter inpatient stays

Whilst individual practitioners can engage with people in trauma-informed ways, this will be inadequate without system-wide changes [45]. In this study, aspects of the nature of the high-secure environment were perceived as barriers to de-escalation. Lack of privacy due to the need for patients to be visible to staff could trigger feelings of shame and promote aggression, despite these ‘lines of sight’ being a necessary requisite to staff and patient safety. Inadequate staffing and few meaningful activities in which to engage were also seen as problematic, as some patients may engage in aggression as a means of relieving boredom. The importance of organisational culture and related staff behaviours was also emphasised. Consistency in maintaining boundaries was particularly emphasized by staff. This clearly important, and the detrimental effects of boundary breaches on both staff and patients in secure mental health settings has been well documented [46, 47]. However, in this study, the function of this practice was framed by staff solely in terms of the perceived need to prevent patients from engaging in what they referred to as ‘pushing boundaries’. This refers to patient efforts to extract concessions or privileges from individual staff members [48]. Notably, patients also valued staff consistency in boundary setting, but largely because they wanted to avoid the consequences of their behaviour being classified by staff as ‘pushing boundaries’. There may be a need for greater critical reflection on which and whose needs boundaries imposed on patient behaviours are serving in order to move staff away from framing this practice in purely adversarial terms.

Some participants felt that some staff members were preoccupied with maintaining custody and control at the detriment of therapeutic care. Others, in particular PMVA instructors, emphasised their promotion of a culture in which de-escalation and positive risk taking is the norm. These findings reflect the tension between the need to promote recovery in high-secure services whilst maintaining safety and security [49].

No qualitative differences in staff attitudes towards de-escalation topics (or other issues raised in focus groups) were found between staff with significant clinical experience (+ 21 years) compared to those with less experience. This is in line with findings that continued exposure to overt aggression causes trauma and fear for both newly-qualified and experienced forensic mental health staff [50, 51] and that similar emotions occur in response to vicarious aggression, such as knowledge of a patient’s offending history [52]. This may suggest that de-escalation approaches may be similar for staff with varying amounts of clinical experience.

In accordance with these findings, all groups of participants highlighted the potential for fear and anxiety to be generated in high-secure hospitals and noted the impact this could have on de-escalation efforts (staff) and receptiveness (patients). Staff fears may result in a reluctance to engage with de-escalation techniques and an overreliance on restrictive practices, increasing the potential for physical and/or psychological harm to those involved. This study, as others [53, 54], highlights the importance of post-incident debriefing on emotional regulation for staff. Patient debriefs were not discussed, perhaps because they are not routinely used, but may minimise negative emotions in patients and mitigate against the breakdown of therapeutic staff–patient relationships [55, 56]. Staff and patient post-incident debriefs may therefore serve as a crucial forum to promote healthy coping strategies, buffer against (re)traumatisation and fear and increase the effectiveness of de-escalation.

Feeling unsafe could cause patients to resort to aggression; a calm reaction by staff was felt to mitigate this. Calmness conveys that the staff member is in control of the situation, whereas fear and nervousness can increase anxiety, make the patient feel unsafe or even that they have gained the ‘upper hand’ [57, 58]. However, staff reported difficulty in maintaining calmness in the face aggression which was personalised towards them—an issue which should be addressed during de-escalation training.

## Strengths and limitations

This in-depth study is the first to give voice though qualitative interviews to high-secure hospital patients and carers regarding the management of violence and aggression. The views described here are of the staff, carers and male patients of one UK-based facility only and may not fully represent those of the wider population. In particular, because the setting was a single institution, the views of women patients were not included; in the UK, however, there are only 50 commissioned female high-secure beds (in one hospital), so female patients constitute a small proportion of the overall high-secure population. Nevertheless, similarities with findings from other forensic settings have been identified and barriers and facilitators specific to the high-secure environment have been highlighted. Recruitment of carers was particularly challenging with only four volunteering to take part. This experience is common to studies in mental health settings [59, 60] and creative ways of accessing carers are needed to ensure that their views are heard. A further strength of the study is the use of the TDF [27] which provided a systematic, evidence-informed approach to identifying barriers and facilitators so that they can be addressed in a future, theory-underpinned intervention. The focus on specific conflict behaviours and behaviour change domains may have helped identify a fuller range of barriers and facilitators than those which might have simply been recalled. Nevertheless, it is possible that some unknown domains not included in the framework may have been missed, though our use of a semi-structured approach to interviews and focus groups was designed to mitigate this.

## Conclusions

For successful de-escalation in a high-secure hospital, both staff and patients need to have the capability to develop strong therapeutic relationships. Knowledge and understanding of the relationship between trauma, the experience of which is especially prevalent among these patients, and aggression is likely to help. Staff and patients have opportunity to develop and apply psychological and interpersonal skills due to the extended stays typical in high-secure settings, however a culture of trust, fairness and consistency is needed to facilitate this. Finally, the potential for and occurrence of violence in high-secure hospitals is high and leads to fear in patients and staff which can inhibit de-escalation attempts. Emotional regulation is important for de-escalation and can be facilitated by post-incident debriefs. Staff and patients are motivated to be safe, so the factors which promote fear in each group should be addressed in de-escalation training.

The findings of this study, and others in different settings, have contributed to the development of an evidence-based, theory informed de-escalation training package which is currently being implemented in ten adult psychiatric wards (acute, PICU and forensic) across England as part of a wider NIHR-funded project (HTA Project: 16/101/02). The feasibility of the training package to improve de-escalation practice is currently being investigated along with the individual, organisational and systemic factors inhibiting or enabling its implementation.

## Supplementary information

**Additional file 1.** Staff topic guide. Topic guide used for PMVA, frontline-clinical and MDT staff focus groups.

**Additional file 2.** Carer topic guide. Topic guide used for carer interviews.

**Additional file 3.** Patient topic guide. Topic guide used for patient interviews.

## Data Availability

Verbatim interview and focus group transcripts are securely stored in the site file at the participating high secure hospital. Due to the potential for indirect patient recognition due to the high-profile nature of this patient subgroup, transcripts and datasets generated during the current study are not publicly available but are available from the corresponding author upon reasonable request.

## Abbreviations

ACE: Adverse childhood event
COM-B: ‘Capability’, ‘opportunity’, ‘motivation’ and ‘behaviour’ model
CPA: Care Programme Approach
EDITION: Enhancing de-escalation techniques in adult acute and forensic units: development and evaluation of an evidence-based training intervention
MDT: Multi Disciplinary Team
NICE: National Institute for Health and Care Excellence
PMVA: Prevention and Management of Violence and Aggression
PRN medication: “Pro Re Nata” (Latin) meaning “as needed”
TDF: Theoretical Domains Framework
