# Supplementary material for: Barriers and facilitators to the effective de-escalation of conflict behaviours in forensic high-secure settings: a qualitative study
Source: Int J Ment Health Syst. 2020 Aug 2;14:59. doi: 10.1186/s13033-020-00392-5 (PMC7397665; doi:10.1186/s13033-020-00392-5)
Supplement: Supplementary file 1 — Additional file 1. Staff topic guide. Topic guide used for PMVA, frontline-clinical and MDT staff focus groups. [file 13033_2020_392_MOESM1_ESM.docx]

**Staff topic guide**

**Introduction**

Thank you for agreeing to take part in this interview/focus group.

Our study aims to develop evidence-based de-escalation training.

For the purposes of this study we define ‘de-escalation’ as the use of verbal and nonverbal skills and strategies to safely reduce unsafe patient behaviours (e.g. self-harm, aggression) without the use of containment interventions (e.g. restraint, PRN, seclusion).

Understanding staff experiences of de-escalation and de-escalation training will be central to the project.

**The interview/focus groups will last a maximum of 1 hour 30 minutes** and cover three topics:

1) Understanding factors that make it easy or hard for staff to help patients to feel calmer when they are feeling distressed, angry, agitated or aggressive/violent.

2) Understanding how staff communicate with patients in response to aggression, self-harm, medication-refusal, suspected drug and alcohol use, rule-breaking and attempts to escape.

3) Your views on optimal de-escalation training including content, delivery, duration and evaluation.

We would like to remind you that everything shared here today will be confidential, unless it relates to someone being put at risk of harm – in these situations, we may need to pass this information on to a member of staff. We will anonymise all data from recordings and transcripts of the group. If you prefer, you can use a pseudonym instead of your real name to introduce yourself to the group. When we write reports on this study it will not contain any names or personal identifiers but we may use anonymous quotes. We would also like to remind everyone to respect the privacy of your fellow participants and not repeat what is said in the focus group to others outside of this room.

Do you have any questions before we begin?

***Topic 1: Understanding how staff communicate with patients in response to aggression, self-harm, medication-refusal, suspected drug and alcohol use, rule-breaking and attempts to abscond.***

- Please tell us about your experiences of effective and ineffective de-escalation of the following patient behaviours:
  - Patient aggression
  - Patient self-harm
  - Medication refusal
  - Attempts to abscond
  - Rule breaking
  - Illicit drug and alcohol use
- How can staff avoid provoking patient aggression or use of physical restraint, enforced medicines or seclusion in response to the above?

***Topic 2: Understanding factors that make it easy or hard for staff to help patients to feel calmer when they are feeling distressed or angry***

- Please outline the essential knowledge staff must have to effectively de-escalate distress/anger/aggression.
  - - What important knowledge gaps have you identified?
    - How should these knowledge gaps be addressed?
  - Which staff emotions are helpful/unhelpful in response to aggression
    - Frightened/emotionless/stressed/burnout/depressed
- What would help improve staff’s emotional state during de-escalation?
- Are there any types of staff that are better at helping patients during these times and why is this?
  - - Qualified vs unqualified/permanent vs temporary
    - What could be done to address skills-deficits among these groups?
    - Are there aspects of professional identity, professional role or professional boundaries that limit the range of de-escalation skills available to you?
  - What factors contribute to effective decision-making in relation to de-escalation? (e.g. when to intervene, how long to de-escalate)
    - What could be done to maximise/support effective decision-making?
  - Please tell us about the clinical systems in place to support de-escalation (assessment, planning, observation, debriefing)
    - What could be done in terms of modifying existing/developing new clinical systems to enhance de-escalation?
  - Are there any patient factors that make it difficult for staff to help them when they are angry/distressed?
    - Illnesses/symptoms, age/gender/cultural background
  - Do different causes of distress affect staff’s ability to calm situations?
    - Which are most difficult for staff to help with? (e.g. Patient-to-patient conflict/being detained)
  - Are there times where it is difficult for staff to calm patients without using restraint, seclusion and medication?
    - How can de-escalation techniques be optimised for these situations?
- What can managers do to support staff to improve use of de-escalation?

o What can they do to reward good practice in terms of the management of distress/anger

o What could they do to reduce poor practice in terms of the management of distress/anger

- Please tell us about team cultures that help/hinder effective use of de-escalation.
  - - Team work/values/ethos/function of team
  - What could be done to foster team cultures that support effective de-escalation?
- Is there anything about the ward environment that can be helpful or unhelpful in making patients feel calmer when they are angry/distressed?
  - How could the environment be improved?
    - Layout/available rooms/décor/calming features/comforting items/equipment
- What goals should hospitals have in terms of increasing non-physical approaches to managing patient distress and reducing use of physical restraint, seclusion and enforced medication?
  - What would help the hospitals to achieve this goal
- Please describe any potential negative consequences of increasing non-physical management of patient anger/distress.
  - How might these be avoided?

***Topic 3: Your views as to how staff should be trained to communicate with patients when they are distressed or angry.***

- Please briefly describe your current de-escalation training and what, if anything, you think is missing from it.
- Please tell us about the most important issues the training should address in terms of improving de-escalation.
- Please tell us how this training should be given to staff – what would help to increase the impact of the training? What would be most helpful in terms of increasing staff to understanding of patient experiences?
- How long should training last?
- How should the training be evaluated – what would count as success?
