# Supplementary material for: Barriers and facilitators to the effective de-escalation of conflict behaviours in forensic high-secure settings: a qualitative study
Source: Int J Ment Health Syst. 2020 Aug 2;14:59. doi: 10.1186/s13033-020-00392-5 (PMC7397665; doi:10.1186/s13033-020-00392-5)
Supplement: Supplementary file 2 — Additional file 2. Carer topic guide. Topic guide used for carer interviews. [file 13033_2020_392_MOESM2_ESM.docx]

**Carer topic guide**

**Introduction**

Thank you for agreeing to take part in this interview/focus group.

Our study aims to develop new training to improve the way staff communicate with patients when they are feeling distressed, angry or aggressive. We hope this will help to reduce unsafe events such as violence, self-harm, physical restraint and seclusion. Exploring carers’ views of the experiences of the people they care for/about will be key to achieving this aim. **The interview/focus groups will take a maximum of 1 hour and 30 minutes** and cover three topics:

1) Exploring views on factors that make it easy or hard for staff to help patients to feel calmer when they are feeling distressed, angry, agitated or aggressive/violent.

2) Understanding how staff communicate with patients in response to aggression, self-harm, medication-refusal, suspected drug and alcohol use, rule-breaking and attempts to escape.

3) Your views as to how staff should be trained to communicate with patients when they are feeling distressed, angry or aggressive.

We would like to remind you that everything shared here today will be confidential, unless it relates to someone being put at risk of harm – in these situations, we may need to pass this information on to a member of staff. We will anonymise all data from recordings and transcripts of the group. If you prefer, you can use a pseudonym instead of your real name to introduce yourself to the group. When we write reports on this study it will not contain any names or personal identifiers but we may use anonymous quotes. We would also like to remind everyone to respect the privacy of your fellow participants and not repeat what is said in the focus group to others outside of this room.

Do you have any questions before we begin?

***Topic 1: Understanding how staff communicate with patients in response to aggression, self-harm, medication-refusal, suspected drug and alcohol use, rule-breaking and attempts to abscond (escape).***

- Please tell us about helpful and unhelpful staff responses to:
  - Patient aggression
  - Patient self-harm
  - Medication refusal
  - Attempts to abscond
  - Rule breaking
  - Illicit drug and alcohol use
- How can staff avoid provoking patient aggression or use of physical restraint, enforced medicines or seclusion in response to the above?

***Topic 2: Understanding factors that make it easy or hard for staff to help patients to feel calmer when they are feeling distressed or angry***

- Please think about times when your relative/person you care for has been angry and/or distressed.
  - What is important for staff to understand about your relative when they are feeling this way?
    - How could staff understanding be improved?
  - Which staff emotions do you think are helpful/unhelpful in these situations?
    - Frightened/emotionless/stressed/burnout/depressed
- What would help improve staff’s emotional state?
- Are there any types of staff that are better at helping your relative and other patients in these situations and why is this?
  - - Qualified vs unqualified/permanent vs temporary
- Are there any patient factors that make it difficult for staff to help them when they are angry/distressed?
  - - Illnesses/symptoms, age/gender
- Do different causes of distress affect staff’s ability to calm situations?
  - Which ones are most difficult for staff to help with?
    - Patient-to-patient conflict/being detained
- Are there times where it is difficult for staff to calm your relative and/or other patients without using restraint, seclusion and medication?
  - - How these situations be managed safely?
- What can managers do to support staff to improve use of de-escalation?
  - What can they do to reward good practice in terms of the management of distress/anger
  - What could they do to reduce poor practice in terms of the management of distress/anger
- Have there been any particular teams that have been helpful/unhelpful in making patients feel calmer when they were distressed/angry
  - What did they do that made them helpful/unhelpful
    - Team work/values/ethos/function of team
  - What could be done to improve responses to patients at a team level?
- Is there anything about the ward environment that can be helpful or unhelpful in making patients feel calmer when they are angry/distressed?
  - How could the environment be improved?
    - Layout/available rooms/décor/calming features/comforting items
- What goals should hospitals have in terms of increasing non-physical approaches to managing patient distress and reducing use of physical restraint, seclusion and enforced medication?
  - What would help the hospitals to achieve this goal
- Please describe any potential negative consequences of increasing non-physical management of patient anger/distress.
  - How might these be avoided?

***Topic 3: Your views as to how staff should be trained to communicate with patients when they are distressed or angry.***

- Please tell us about the most important issues the training should address in terms of improving staff responses to patient distress or anger?
- Please tell us how this training should be given to staff – what would help to increase the impact of the training? What would be most helpful in terms of increasing staff understanding of patient experiences?
- How long should training last?
- How should the training be evaluated – what would count as success?
